# Impact of Xpert^®^ Carba-R-based screening for carbapenem-resistant organisms on infection-related mortality in hematopoietic stem cell transplant recipients

**DOI:** 10.3389/fcimb.2025.1698525

**Published:** 2025-12-17

**Authors:** Chenjing Qian, Jiaxin Hong, Wei Shi, Fang Liu, Weiming Li, Ling Ma, Xinghui Gao, Yi-Wei Tang, Qiuling Wu, Linghui Xia, Mei Hong

**Affiliations:** 1 Institute of Hematology, Union Hospital, Tongji Medical College, Huazhong University of Science and Technology, Wuhan, Hubei, China; 2 Blood Disease Transplantation and Immunology Key Laboratory, The First Affiliated Hospital of Henan University of Science and Technology, Luoyang, Henan, China; 3 Cancer Center, Union Hospital, Tongji Medical College, Huazhong University of Science and Technology, Wuhan,, China; 4 Department of Clinical Laboratory, Union Hospital, Tongji Medical College, Huazhong University of Science and Technology, Wuhan, China; 5 Medical Affairs, Danaher Corporation/Cepheid China, Shanghai, China; 6 College of Public Health, Chongqing Medical University, Chongqing, China; 7 Collaborative Innovation Center of Hematology, Soochow University, Suzhou, China

**Keywords:** carbapenem-resistant organisms, HSCT, colonization, infection, Xpert Carba-R assay

## Abstract

**Background:**

The emergence of carbapenem-resistant organisms (CROs) poses a major challenge to clinical infection control in hospitals. Patients undergoing hematopoietic stem cell transplants (HSCTs) infected with CROs are at high risk of mortality. Proactive screening of HSCT patients for CRO colonization may enable early and accurate preemptive anti-CRO therapy, reduce the probability of secondary infections, and contribute to infection prevention and control measures. However, screening CRO colonization with stool/rectal swab culture and sensitivity has a low positivity rate with a long turnaround time, which limits the effectiveness of the interventions. A more rapid and accurate method to detect CRO colonization is urgently needed. Xpert Carba-R assay provides a rapid and accurate detection of carbapenemase types, enabling targeted anti-infective therapy selection based on the identified resistance mechanism.

**Methods:**

We conducted a historically controlled prospective study at Union Hospital, Tongji Medical College, Huazhong University of Science and Technology between August 2021 and July 2022. The study population comprised adult HSCT patients (≥18 years old) who received preemptive anti-CRO therapy based on rectal culture and Xpert Carba-R screening during this period. A total of 381 patients who underwent HSCT from August 2020 to July 2022 were included in the study, and CRO colonization screening was performed on admission and weekly thereafter. In the historic control group from August 2020 to July 2021, HSCT patients were screened only by rectal swab traditional CRO culture, and CRO colonization was determined if the rectal culture was positive. In the study group from August 2021 to July 2022, two rectal swab specimens were collected from HSCT patients for both CRO traditional culture (RS-culture) and Xpert Carba-R testing (RS-Carba-R). CRO colonization was determined if either of the screening methods was positive. CRO-active antibiotics were immediately provided on the first febrile episode of neutropenia (FN) in CRO-colonized patients. Clinical outcome data for the CRO monthly colonization rate and anti-infection efficiency were collected and contrasted between the two groups.

**Results:**

In the historic group, 47 out of 197 patients (23.9%) were identified as colonized with CRO, detected only by RS-culture. In the study group, 41 out of 184 patients (22.3%) were identified as colonized with CRO, detected by either RS-culture or RS-Carba-R; among them, *Escherichia coli* was the most common CP-CRO strain, and the most prevalent carbapenemase type was NDM. This indicated a slightly lower annual detection rate of CRO gut colonization in the study group compared to the historic group, but no significant difference was observed (22.3% *vs*. 23.9%, *p* = 0.715). The incidence of CRO-related bloodstream infections (CRO-BSI) was significantly lower in the study group compared to the historic group (4.8% *vs*. 25.5%, *p* = 0.012), and the CRO-related mortality in colonized patients decreased from 19.4% in the historic group to 2.4% in the study group (*p* = 0.046). The monthly detection rate of CRO gut colonization by RS-culture in the historic group remained steady, with no significant fluctuation (19.7% in the first month and 18.8% in the last month). In contrast, the monthly detection rate of CRO gut colonization by either RS-culture or RS-Carba-R in the study group was higher in the first month compared to the historic group (21.2% in August 2021 *vs*. 18.8% in July 2021). However, a gradual decline in the monthly detection rates of CRO gut colonization by RS-culture and/or RS-Carba-R was observed in the study group, dropping from 21.2% in the first month to 2.9% in the last month. The univariate and multivariate analyses indicated that the study group had a shorter length of hospitalization (OR = 0.94, 95% CI 0.88–0.99, *p* = 0.038) and CRO-related mortality (OR = 0.12, 95% CI 0.01–0.75, *p* = 0.021) than the historic group.

**Conclusions:**

Our study showed a positive effect of more rapid CRO colonization screening using rectal swabs with Xpert Carba-R and culture, which can guide potent CRO preemptive therapy for subsequent infections based on the detected carbapenemase mechanism, thereby reducing mortality and the spread of CRO infection in HSCT patients.

**Clinical Trial Registration:**

chictr.org, identifier ChiCTR2100041976.

## 1 Introduction

Recently, the worldwide spread of carbapenem-resistant organisms (CROs), including carbapenem-resistant *Enterobacterales* (CRE), carbapenem-resistant *Acinetobacter baumannii* (CRAB), and carbapenem-resistant *Pseudomonas aeruginosa* (CRPA), has emerged as a significant medical and public health problem, demanding urgent action (Tacconelli et al., 2018; Kois et al., 2020). Patients undergoing hematopoietic stem cell transplantation (HSCT) are especially susceptible to CRO infection due to the receipt of multiple rounds of radiation therapy, high-dose chemotherapy, or immunosuppressive therapy during transplantation. They also have additional risk factors for infection, including mucositis, neutropenia, prolonged hospital stays, and frequent use of prophylactic antimicrobial agents (Trecarichi et al., 2016). Among HSCT patients, the incidence of CRO infection ranges from 8.4% to 10.0% in Europe (Averbuch et al., 2017; Girmenia et al., 2017) and from 6.2% to 10.4% in China (Wang et al., 2015; Ge et al., 2018; Yan et al., 2018), where infections in allo-HSCT patients (15.8% to 23.7%) were higher than in auto-HSCT patients (4.8% to 8.9%). Furthermore, reports show that HSCT patients who developed CRE-related infections have an overall mortality rate of 58% at 3 months, with allo-HSCT patients showing a significantly higher mortality rate than auto-HSCT patients (Averbuch et al., 2017; Girmenia et al., 2017). Because CRO−associated infection has features of occult onset, quick development, and serious complications, despite using aggressive recommended regimens, delayed anti-CRO therapy remains the primary factor of therapeutic failure and early death. In one study, 69% (9/13) of neutropenic CRO-infected patients died with a median of 4 days from presentation until death (Satlin et al., 2013). Early identification of CRO−associated infection is paramount for developing early anti-infective strategies and reducing mortality in transplant patients.

The gut is an important reservoir of both bacterial virulence genes and antimicrobial resistance genes, such as carbapenemase genes. Colonization of the gut with CRO is generally recognized as a prerequisite for CRO infection; thus, CRO-colonized patients are at particularly high risk of subsequent CRO infections during their hospital stay (Tamma et al., 2019). Screening of the gut flora using either rectal swabs or fecal specimens to detect CRO colonization in patients is recommended by the CDC and ECDC to reduce subsequent clinical infections and transmission (Tascini et al., 2014; Davido et al., 2017; Zheng et al., 2020). A recent retrospective observational study (Krupanandan et al., 2023) has shown that active CRO surveillance decreased CRO infection rates in the pediatric intensive care unit (PICU). Rectal swabs for CRO were sent from all PICU patients, and contact isolation precautions were followed for rectal swab-positive patients. Post-intervention, ICU-acquired CRO colonization decreased by 36%, and ICU-acquired CRO infection rates decreased by 100% (*p* < 0.0001). However, screening the gut for CRO has a low positivity rate with a long turnaround time, which limits the effectiveness of the interventions (Asai et al., 2018). Therefore, exploring more rapid and accurate detection methods to detect colonization of the gut with CRO is crucial for the optimization of subsequent CRO infection control measures.

Carbapenem resistance among bacterial species is mediated by heterogeneous mechanisms, including the production of carbapenemases, extended-spectrum β-lactamases, and/or AmpC cephalosporinases, the latter two being frequently combined with active drug efflux or membrane impermeability, culminating in resistance (Livingstone et al., 1995). Carbapenemase production (CP) is the most problematic. The public health threat of CROs is amplified by the fact that many of the most prevalent carbapenemase genes (e.g., those encoding KPC, NDM, and OXA-48-like enzymes) are located on mobile genetic elements such as plasmids, facilitating their horizontal transfer between bacterial species, but others may be chromosomally encoded (e.g., OXA-51 in *Acinetobacter baumannii*) (Kazmierczak et al., 2021). It was reported that patients dying from bacteremia within 14 days of CP-CRO infection were four times greater than non-CP-CRO infection (Tamma et al., 2017). The Xpert Carba-R test (Cepheid, Sunnyvale, CA) is a novel carbapenemase gene detection technique designed to detect the five most common carbapenemase gene families, namely, KPC, NDM, VIM, IMP, and OXA-48, in less than 1 h using multiplex real-time polymerase chain reaction (PCR) (Dortet et al., 2016). The test has demonstrated promising value for the rapid identification of carbapenemase-producing organisms from clinical surveillance specimens (Tato et al., 2016; Moore et al., 2017), but its performance in HSCT patients remains unclear.

In this study, we aimed to investigate the performance of the Xpert Carba-R test versus rectal culture for the detection of gut CRO colonization in HSCT patients and to compare clinical outcomes, including the incidence of subsequent infections, the rates of mortality, and the efficacy of anti-infective therapy for subsequent CRO infections in HSCT patients.

## 2 Materials and methods

### 2.1 Study design and population

This historically controlled prospective study was approved by the Ethics Committee of Union Hospital, Tongji Medical College, Huazhong University of Science and Technology, and registered on chictr.org.cn (ChiCTR2100041976). The collection of specimens from HSCT patients was performed after signing the informed consent. The historically controlled group included patients who received preemptive anti-CRO therapy based on only rectal culture screening from August 2020 to July 2021. The study group included HSCT patients who received preemptive anti-CRO therapy based on rectal culture and Xpert Carba-R screening from August 2021 to July 2022. All patients who were included in the study group and the historic group met the same inclusion criteria as follows: were ≥18 years of age, received a standard-of-care HSCT, and gave informed consent for CRO screening. The exclusion criteria were patients <18 years of age and patients who died due to primary disease, such as cerebral hemorrhage due to thrombocytopenia, or those who died due to infections caused by definite fungal infection or other bacteria. In this study, only the first neutropenic fever (FN) episode was included in each patient. The primary endpoint of the study was attributed mortality at day 30 from the first FN episode, and the secondary endpoints were anti-infective effectivity, including the duration of fever resolution, total duration of hospitalization, effective rate of antibiotic therapy, and mortality. CRO detection rate, CRO-BSI incidence, and CRO-related mortality at day 30 from the positivity of blood cultures were also evaluated. The CRO gut colonization rate and the CRO-BSI rate were evaluated. Case records were analyzed for demographic, clinical, and microbiological details. Of note, all HSCT patients with acute leukemia, lymphoma, multiple myeloma, and myelodysplastic syndrome (MDS) underwent myeloablative or non-myeloablative conditioning regimen. All patients were given a peripherally inserted central catheter (PICC) for the administration of chemotherapy drugs. Prophylactic antibiotics were not used for all patients. Empirical antifungal therapy prophylaxis was administered in HSCT patients, especially in allogeneic recipients, according to current guideline recommendations (Freifeld et al., 2011).

**Figure 1:**
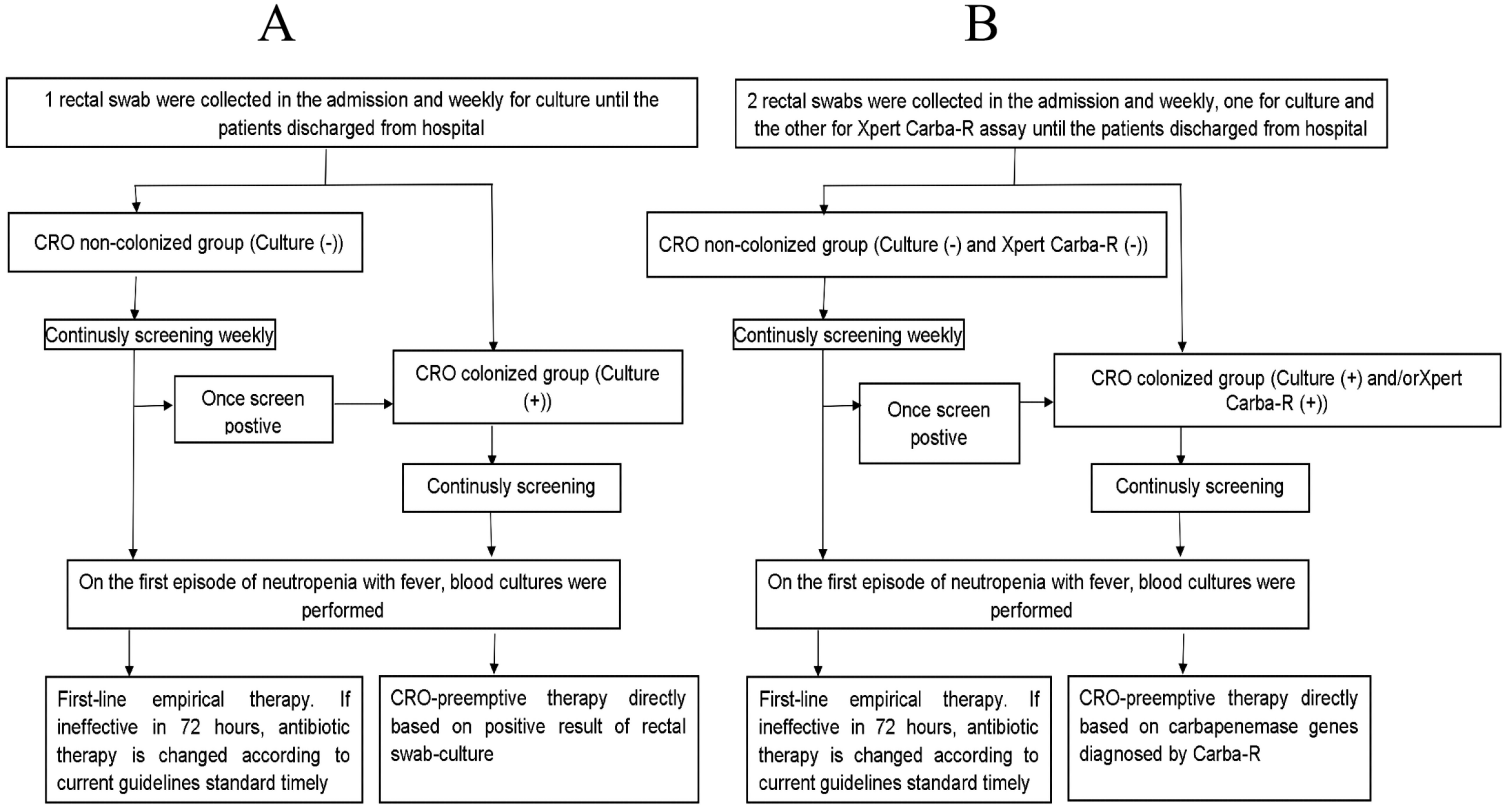
**(A)** Flowchart for the diagnosis and treatment of CRO in HSCT patients (the historic group). **(B)** Flowchart for the diagnosis and treatment of CRO in HSCT patients (the study group).

The study group design is shown in **Figure 1A**. From August 2021 to July 2022, 184 consecutive patients were enrolled in the study group. Among them, rectal swabs for Xpert Carba-R testing were collected on admission and weekly until hematopoietic reconstruction for patients who remained hospitalized. Two rectal swabs per patient were collected simultaneously for traditional CRO culture (RS-culture) and Xpert Carba-R testing (RS-Carba-R). The time to result for both tests was recorded separately. CRO screening was repeated in patients who had fever or had gut complications, such as abdominal pain, diarrhea, and perianal inflammation. Once a positive RS-culture and/or RS-Carba-R was detected, the purified CRO strain was stored in a −80 °C freezer for Carba-R testing later (purified culture Carba-R) to confirm the presence of carbapenemase genes. HSCT patients with either a positive RS-culture and/or positive RS-Carba-R test were determined as CRE colonization, and contact precautions were applied, including a single room, a hanging sign, hand hygiene performed before and after entering the room, use of disposable gloves and gowns, and strengthened environmental cleaning and disinfection (French et al., 2017; WHO Guidelines Approved by the Guidelines Review Committee, 2017). On the first episode of neutropenic (FN) fever in CRO-colonized patients, blood cultures were performed, and anti-CRO preemptive therapy was immediately initiated according to the result of the RS-culture and carbapenemase genes based on immediate RS-Carba-R, including tigecycline, polymyxins, and novel β-lactamase inhibitor combinations (e.g., if KPC+, then ceftazidime-avibactam was given; if NDM+, then ceftazidime-avibactam + aztreonam was given) (Sahitya et al., 2021). The above drugs were given as a two-drug or a three-drug combination according to the actual clinical manifestations of infections and under all the following conditions: i) neutropenic fever persistent over 12 h or fever with obvious symptoms of intestinal infection, such as abdominal pain, diarrhea, and perianal pain; ii) C-reactive protein (CRP) at five times higher than normal with or without a significant increase in procalcitonin (PCT); and iii) signs of progression of sepsis. For FN patients with RS-culture (−) and RS-Carba-R (−), which were defined as the CRO non-colonized group, first-line empirical therapy with either BL BLI, cefoperazone–sulbactam, piperacillin/tazobactam, cefepime, ceftazidime, or carbapenem, including meropenem or imipenem, was initiated according to current guideline recommendations (Freifeld et al., 2011; Flowers et al., 2013). If ineffective in 72 h, antibiotic therapy was changed to step-up therapy according to current guidelines, including fluoroquinolones (ciprofloxacin), aminoglycosides (amikacin), aztreonam, tigecycline, polymyxins (polymyxin B), and combination therapy (Freifeld et al., 2011). A composite adjudication of clinical antimicrobial effectivity and rationality for each FN patient was made independently by a committee composed of three independent board-certified infectious disease physicians according to symptoms and signs, radiological testing results, infection-related laboratory tests, and results of microbiological tests. The rationality of the antimicrobial regimen was evaluated according to the clinical practice guideline for the use of antimicrobial agents in neutropenia patients with cancer (Freifeld et al., 2011). After three consecutive negative rectal swabs, contact precautions were relieved.

**Figure 1B** shows the flowchart of the historic group. The patients in the historic matched control group (*n* = 197) from the previous year (August 2020 to July 2021), who were identified positive for CRO gut colonization only by RS-culture, received CRO preemptive therapy, including tigecycline, polymyxins, and novel β-lactamase inhibitor combinations, such as ceftazidime/avibactam and aztreonam, which was also adopted for subsequent infection in the CRO-colonized group similar to the study group.

### 2.2 Bacterial identification and antimicrobial susceptibility testing

If a patient is neutropenic and febrile, blood cultures were drawn, and 8–10 mL of blood was collected in culture bottles by a healthcare professional following the “two sides, two sets” principle and sent for testing. The isolation and identification of bacteria from the blood and RS specimens were carried out in accordance with the relevant provisions of the National Clinical Laboratory procedures. Bacterial species identification and antimicrobial susceptibility tests were performed using a Vitek^®^ 2 automated system (bioMérieux, France) and matrix-assisted laser-desorption ionization time-of-flight mass spectrometry (Bruker Daltonics Inc., Billerica, Massachusetts, USA). *Escherichia coli* ATCC 25922, *E. coli* ATCC 35218, and *Klebsiella pneumoniae* ATCC 700603 were tested as the quality control strains for antimicrobial susceptibility testing. All antibiotics, except tigecycline and colistin, were interpreted according to the standard of the CLSI M100S 28th document (CLSI, 2020). Carbapenem resistance was defined as an ertapenem MIC ≥2 µg/mL or a meropenem and/or imipenem MIC ≥4 µg/mL. Identification of drug resistance was carried out both by the disk diffusion (K-B) method and broth microdilution (BMD), with BMD used to determine MICs. For tigecycline and colistin, antimicrobial susceptibility testing was performed and interpreted according to the European Committee on Antimicrobial Susceptibility Testing (EUCAST) clinical breakpoint guidelines, version 10.0 (2020) (Åkerlund et al., 2020).

### 2.3 Xpert Carba-R

Carbapenemase genes (KPC, NDM, VIM, IMP, OXA-48-type) were detected using the Xpert Carba-R assay on the GeneXpert system (Cepheid, USA). Following the manufacturer’s protocol, rectal swab specimens or purified colonies were suspended in the provided buffer, loaded into the cartridge, and processed by the instrument for automated DNA extraction, amplification, and detection.

### 2.4 Definitions

Neutropenia was defined as a neutrophil count <500/mL or an expectation that a patient would be neutropenic within 48 h (Freifeld et al., 2011). FN was defined as a fever episode (axillary temperature—37.5 °C or higher—was measured in all patients) during neutropenia (Okamoto et al., 2021). The resolution of fever was defined as an axillary temperature <37.5°C maintained for 72 consecutive hours after antibiotic therapy.

CRO-BSI was defined as a BSI documented by blood culture positivity (at least one specimen) for a CRO strain and clinical signs of systemic inflammatory response syndrome.

Effective antibiotic therapy, including effective CRO preemptive therapy, was defined as the patient achieving resolution of fever, abatement of inflammatory markers (C-reactive protein, procalcitonin), and resolution of any clinical symptoms compatible with an infection within a maximum of 72–96 h after initiation of antibiotic therapy (Schmidt-Hieber et al., 2019).

Total length of hospitalization refers to the number of days transplanted patients spent in laminar airflow rooms from the day of transplantation until discharge. The deaths caused by evident fungal infection were excluded from the calculation of mortality.

### 2.5 Statistical analysis

Continuous variables were compared using the Student’s *t*-test (for normally distributed variables) or the Mann–Whitney *U* test (for non-normally distributed variables) and presented as the mean ± standard deviation (SD) or median. Categorical variables were assessed using the *χ*^2^ test or Fisher’s exact test and presented as percentages. Logistic regression (backward LR) methods (univariate, multivariate) were used to identify independent factors between the historic group and the study group. Odds ratios (ORs) and their corresponding 95% confidence intervals (CIs) were calculated. A two-tailed *P*-value of <0.05 was considered statistically significant. All statistical analyses were performed using the SPSS software, version 25.0 (SPSS Inc., Chicago, Illinois).

## 3 Results

### 3.1 Patient characteristics and detection of CRO colonization

**Table 1** shows the demographic and clinical characteristics of the patients. A total of 381 patients who underwent HSCT from August 2020 to July 2022 were included in the study. There were 215 male patients (56.4%) and 166 female patients (43.6%). In terms of disease distribution, acute myelocytic leukemia (AML) was the predominant primary disease. There were 14.2% patients who underwent autologous HSCT (54 out of 381) and 85.5% patients who underwent allogeneic HSCT (327 out of 381), and among the allogeneic recipients, 71.7% received grafts from HLA non-identical donors (235 out of 327) and 28.3% from HLA identical donors. In both groups, the predominant pathogen of CRO gut colonization was *E. coli*. Factors including gender, age, underlying disease, type of HSCT, gender of the donor and recipient, blood type of the donor and recipient, HLA type, detection rates of CRO gut colonization, and type of CRO gut colonization were not statistically significant between the study group and the control group. In the historic group, a total of 13 patients died (13/197, 6.6%), and 184 patients survived, while in the study group, 2 patients died (2/184, 1.1%), and 182 patients survived.

**Table 1:** Comparison of baseline characteristics of HSCT patients in the intervention and control groups.

Metrics		Historic group *N* = 197 (%)	Study group *N* = 184 (%)	*p*
Characteristics				
	Gender			0.739
	Male	110 (55.8%)	105 (57.1%)	
	Female	87 (44.2%)	79 (42.9%)	
	Age			0.207
	≤45 years	144 (73.1%)	123 (66.8%)	
	45–60 years	50 (25.4%)	56 (30.4%)	
	≥60 years	3 (1.5%)	5 (2.8%)	
Primary disease				0.443
	AML	64 (32.5%)	60 (32.6%)	
	ALL	54 (27.4%)	40 (21.7%)	
	SAA	23 (11.7%)	25 (13.6%)	
	MM	22 (11.2%)	22 (12.0%)	
	MDS	13 (6.6%)	17 (9.2%)	
	Lymphoma	11 (5.5%)	13 (7.1%)	
	Others	10 (5.1%)	7 (3.8%)	
HSCT				0.274
	Autologous	32 (16.2%)	22 (12.0%)	
	Allogenic	165 (83.8%)	162 (88.0%)	
Gender of the donor and recipient				0.133
	Compatible	91(46.2%)	71 (38.6%)	
	Incompatible	106(53.8%)	113 (61.4%)	
Blood type of the donor and recipient				0.102
	Compatible	116(58.8%)	93 (50.5%)	
	Incompatible	81(41.2%)	91 (49.5%)	
HLA				0.073
	Identical	84(42.6%)	62 (33.7%)	
	Non-identical	113(57.4%)	122 (66.3%)	
RS-culture				0.161
	Positive	47 (23.6%)	36 (19.6%)	
	Negative	150 (76.4%)	148 (80.4%)	
Parameters				
Max temperature (°C)		38.83 ± 0.63	38.66 ± 0.58	0.276
Max CRP (mg/L)		129.35 ± 49.73	119.44 ± 49.90	0.374
Duration of G-CSF used (days)		9.05 ± 4.11	8.28 ± 4.25	0.100
Duration of FN (days)		11.06 ± 4.14	10.28 ± 4.28	0.102
Duration of recovery from neutropenia (days)		7.05 ± 4.13	6.27 ± 4.27	0.112
Duration of fever resolution		3.72 ± 2.27	3.14 ± 1.59	0.025
Duration of hospitalization		34.18 ± 9.36	28.32 ± 5.83	0.010
Antibiotic therapy after the first FN episode				0.045
	Effective	112 (56.9%)	123 (66.8%)	
	Ineffective	85 (43.1%)	61 (33.2%)	
Survival status				<0.001
	Survival	184 (93.4%)	181 (98.9%)	
	Death	13 (6.6%)	2 (1.1%)	

AML, acute myeloblastic leukemia; ALL, acute lymphoblastic leukemia; SAA, mixed phenotype acute leukemia, severe aplastic anemia; MM, multiple myeloma; MDS, myelodysplastic syndrome; HSCT, hematopoietic stem cell transplantation; HLA, human leukocyte antigen; CRP, C-reactive protein; G-CSF, granulocyte colony-stimulating factor; FN, febrile neutropenia; neutropenia, ANC <0.5 × 10^9^/L.

### 3.2 CRO-BSI incidence and clinical outcomes

In the historic group, 47 patients (47/197, 23.9%) were identified as colonized with CRO determined by the RS-culture, and 12 of these patients developed confirmed CRO-BSI (12/47, 25.5%). Ten out of the 12 patients died, and 9 patients died of CRO-related septic shock (9/47, 19.4%). In the study group, 41 (22.3%) out of 184 patients were identified to be colonized with CRO determined by the RS-culture and/or RS-Carba-R. Of these patients, two later developed confirmed CRO-BSI (2/41, 4.8%) during the HSCT period, and then one died of CRO-related septic shock (1/41, 2.4%). Due to the introduction of the immediate RS-Carba-R continuous screening in the study group, the annual detection rate (calculated as the number of new CRO-positive patients identified per year divided by the total number of patients screened that year) of CRO gut colonization has a lower percentage than that in the historic group (22.3% *vs*. 23.9%, *p* = 0.715), and the morbidity of CRO-BSI showed a significantly decreased percentage than that in the historic group (4.8% *vs*. 25.5%, *p* = 0.012). The CRO infection-related mortality in the CRO-colonized patients dropped from 19.4% in the historic group to 2.4% in the study group (*p* = 0.046).

### 3.3 CRO isolate strains and carbapenem-resistant genes in CRO gut colonization

**Figure 2:**
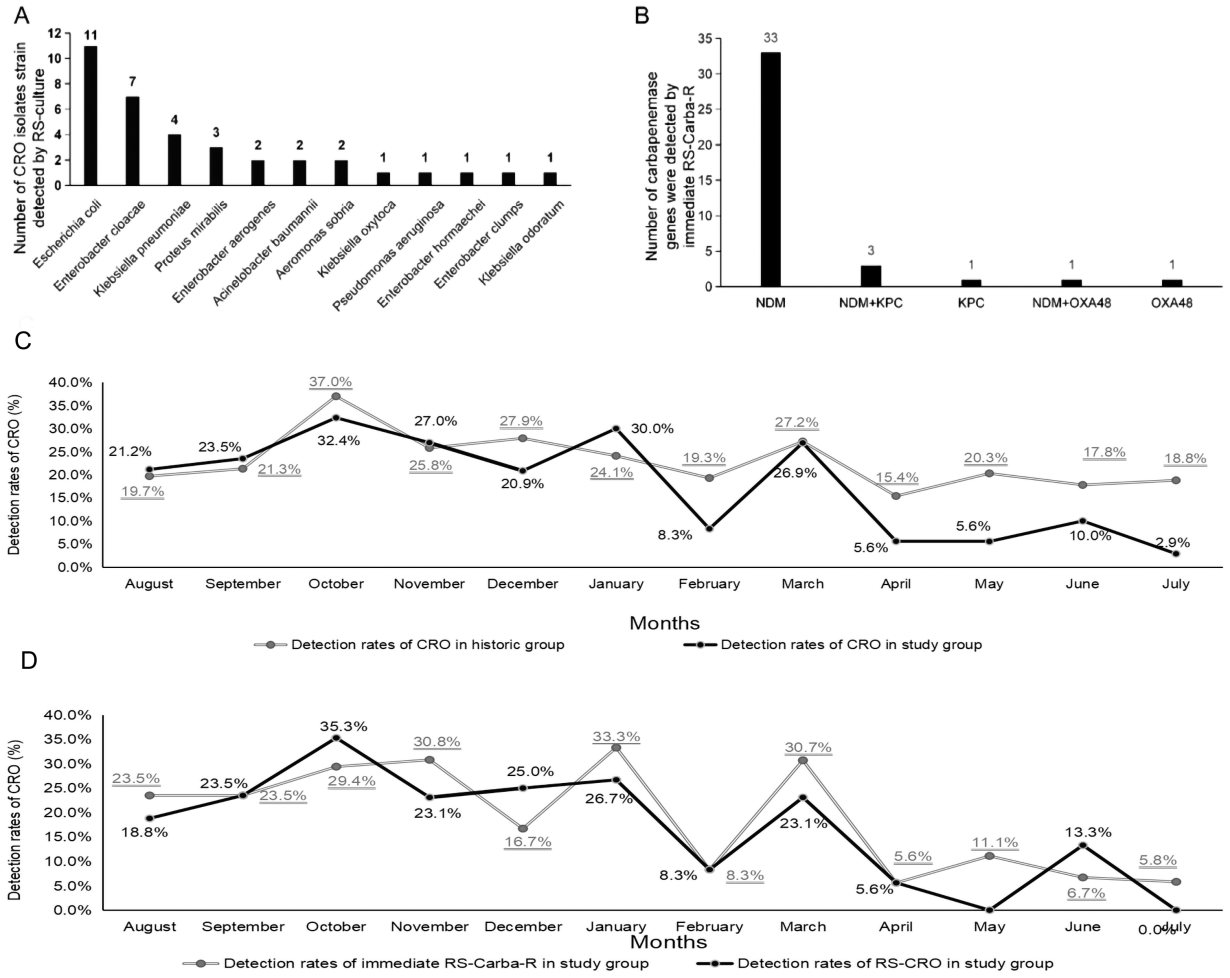
**(A)** Distribution of CRO bacterial species detected by rectal swab culture in the study group. **(B)** Distribution of carbapenemase genes detected by the immediate RS-Carba-R assay in the study group. **(C)** Monthly detection rate of CRO colonization.

As shown in **Figure 2A**, a total of 36 CRO isolate strains were detected by the RS-culture in the study group, with the top strain being *E. coli* (11/36, 30.6%), and a total of 39 carbapenem-resistant genes were detected by immediate RS-Carba-R in the study group (**Figure 2B**). The most frequently detected carbapenem-resistant gene was NDM (33/39, 84.6%). VIM and IMP genotypes were not detected by the Xpert Carba-R assay in the study group.

### 3.4 The monthly CRO detection rate of CRO gut colonization

As shown in **Figure 2C**, the monthly detection rate of CRO gut colonization determined by the RS-culture in the historic group from August 2020 to July 2021 was recorded (gray polyline), and the monthly detection rate of CRO gut colonization determined by the RS-culture and/or RS-Carba-R in the study group from August 2021 to July 2022 was also recorded (black polyline). The monthly detection rate of CRO gut colonization determined by the RS-culture in the historic group remained steady without significant fluctuation (19.7% in the first month and 18.8% in the last month). The last monthly detection rate of CRO gut colonization determined by the RS-culture in the historic group was lower than the first monthly detection rate of CRO gut colonization determined by RS-culture and/or RS-Carba-R in the study group (18.8% in July 2021 and 21.2% in August 2021) due to the introduction of the sensitive Xpert Carba-R. However, the monthly detection rate of CRO gut colonization determined by the RS-culture and/or RS-Carba-R gradually declined in the study group (21.2% in the first month and 2.9% in the last month). Moreover, in the study group, a similar decreasing tendency in the monthly detection rates of CRO gut colonization was found as determined by the RS-culture (18.8% in the first month and 0% in the last month) (black polyline, **Figure 2D**) and RS-Carba-R (23.5% in the first month and 5.8% in the last month) (gray polyline, **Figure 2D**), separately. We believed that the most plausible explanation was the earlier implementation of infection control measures made possible by the rapid turnaround time of the Xpert^®^ Carba-R assay, which can guide potent CRO preemptive therapy of subsequent infection based on the detected carbapenemase mechanism. This allowed for the prompt isolation of CRO carriers, potentially reducing nosocomial transmission.

### 3.5 The concordance of carbapenemase genes detected by immediate RS-Carba-R and purified colony Carba-R in the same patient

In the study group of 184 patients, we used Xpert Carba-R to detect the carbapenemase gene types in real-time rectal swab specimens from the patients, referred to as immediate RS-Carba-R. Additionally, we applied Xpert Carba-R to detect the carbapenemase genes carried by CRO isolates, which were cultured from traditional rectal swabs collected from the same patient at the same time, referred to as purified colony Carba-R. We compared the consistency of the carbapenemase gene detection results between immediate RS-Carba-R and purified colony Carba-R from the same patient at the same time, with the findings recorded in **Table 2**. *Escherichia coli* (*n* = 11) was the most frequently detected CRO isolate strain by the RS-culture, and among them, the most common resistance gene detected in both purified colony Carba-R and immediate RS-Carba-R was NDM (*n* = 9). The resistance genes detected by purified colony Carba-R were consistent with immediate RS-Carba-R in 81.8% (9/11) of *E. coli* cases. One *E. coli* strain carried both NDM and KPC as identified by purified colony Carba-R, while NDM+OXA-48 identified by immediate RS-Carba-R was detected in the same patient, which implies that immediate RS-Carba-R has higher sensitivity, as a potential OXA-48-carrying bacterium might not have been detected by culture. Furthermore, another *E. coli* strain carried the KPC resistance gene as detected by purified colony Carba-R, but no resistance gene was identified by immediate RS-Carba-R in the same patient, and this could have been due to a sampling error; however, a subsequent repeat rectal swab tested with the immediate RS-Carba-R assay detected the same KPC enzyme. For *K. pneumoniae* (*n* = 4), two strains carried KPC and one strain carried both NDM and KPC, as detected by purified colony Carba-R. However, all four strains carried both NDM and KPC when detected by immediate RS-Carba-R in the same patient. In another case, *K. pneumoniae* carried NDM, as detected by purified colony Carba-R, but immediate RS-Carba-R identified KPC in the same patient. The carbapenemase genes detected by purified colony Carba-R were consistent with those detected by immediate RS-Carba-R in only 50% (2/4) of *K. pneumoniae* isolates. As for *Proteus mirabilis* (*n* = 3), two strains carried NDM, as detected by both purified colony Carba-R and immediate RS-Carba-R in the same patient. One strain carried both NDM and OXA-48, as detected by purified colony Carba-R, but only OXA-48 was detected by immediate RS-Carba-R in the same patient. Therefore, carbapenemase gene detection by purified colony Carba-R was consistent with immediate RS-Carba-R in 66.7% (2/3) of *P. mirabilis* cases. For other CRO strains, including *Enterobacter cloacae* (*n* = 7), *Enterobacter aerogenes* (*n* = 2), *Klebsiella oxytoca* (*n* = 1), *Enterobacter hormaechei* (*n* = 1), and *Enterobacter clumps*, the resistance genes detected by purified colony Carba-R were 100% consistent with immediate RS-Carba-R in the same patient. No resistance genes were detected by either immediate RS-Carba-R or purified colony Carba-R in the CRO strains *Acinetobacter baumannii* (*n* = 2), *Aeromonas sobria* (*n* = 2), *Pseudomonas aeruginosa* (*n* = 1), and *Klebsiella odoratum* (*n* = 1). Additionally, 11 patients were tested for NDM resistance genes using immediate RS-Carba-R, but no CRO species were detected by the RS-culture in these cases.

**Table 2:** Agreement in carbapenemase gene detection by direct rectal swab versus bacterial colony isolate using the Xpert Carba-R assay.

CRO isolate strains detected by the RS-culture (*N*)	Carbapenemase gene detected by purified colony Carba-R (*N*)	Carbapenemase gene detected by immediate RS-Carba-R (*N*)	Concordance rate
*Escherichia coli* (11)	NDM (9), KPC (1), NDM+KPC (1)	NDM (9), NDM+OXA-48 (1), NA (1)	81.8%
*Enterobacter cloacae* (7)	NDM (6), NA (1)	NDM (6), NA (1)	100%
*Klebsiella pneumoniae* (4)	KPC (2), NDM+KPC (1), NDM (1)	NDM+KPC (3), KPC (1)	50%
*Proteus mirabilis* (3)	NDM (2), NDM+OXA-48 (1)	NDM (2), OXA-48 (1)	66.7%
*Enterobacter aerogenes* (2)	NDM (2)	NDM (2)	100%
*Acinetobacter baumannii* (2)	NA (2)	NA (2)	100%
*Aeromonas sobria* (2)	NA (2)	NA (2)	100%
*Klebsiella oxytoca* (1)	NDM (1)	NDM (1)	100%
*Pseudomonas aeruginosa* (1)	NA (1)	NA (1)	100%
*Enterobacter hormaechei* (1)	NDM (1)	NDM (1)	100%
*Enterobacter* clumps (1)	NDM (1)	NDM (1)	100%
*Klebsiella odoratum* (1)	NA (1)	NA (1)	100%
None detected (11)	NA (11)	NDM (11)	0

### 3.6 Therapeutic evaluation of RS-Carba-R-guided therapy

To evaluate the performance of RS-Carba-R in guiding the antibiotic treatment in CRO-colonized patients when they suffered from subsequent infection, we compared the anti-infective effectivity, including the duration of fever resolution, total duration of hospitalization, the effective rate of antibiotic therapy, and mortality, between the study group with CRO colonization surveillance by RS-Carba-R and RS-culture and the historic group with CRO colonization surveillance by only RS-culture.

**Figure 3:**
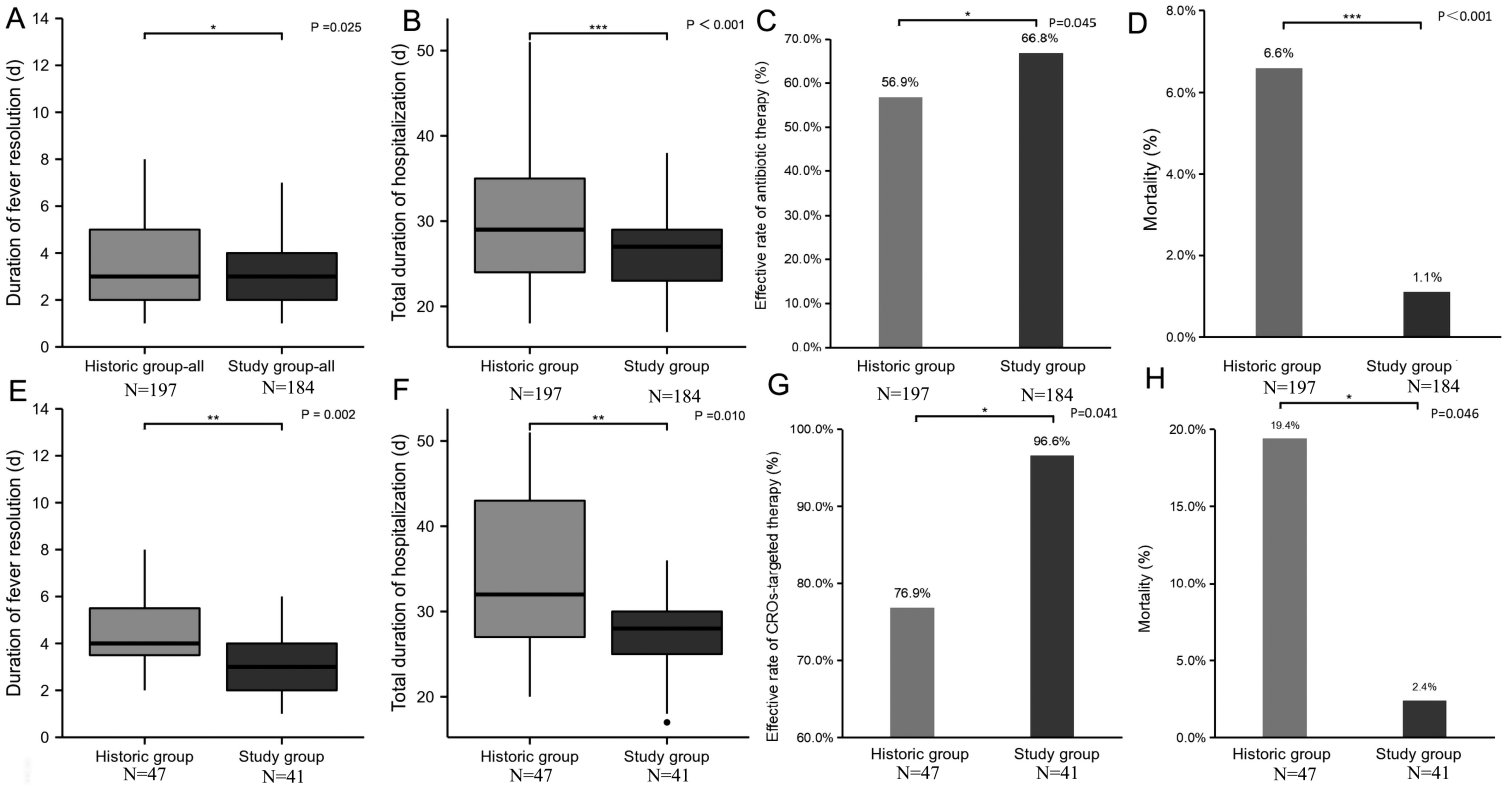
**(A–D)** The anti-infective effectivity in all HSCT patients in the study group: **(A)** duration of fever resolution in all HSCT patients: the historic group *vs*. the study group; **(B)** total hospitalization duration in all HSCT patients: the historic group *vs*. the study group; **(C)** antibiotic therapy effective rate after the first febrile neutropenia episode in all HSCT patients: the historic group *vs*. the study group; and **(D)** mortality rate in all HSCT patients: the historic group *vs*. the study group. **(E–H)** The anti-infective effectivity in all HSCT patients in the study group: **(E)** duration of fever resolution in CRO-colonized HSCT patients: the historic group *vs*. the study group; **(F)** total hospitalization duration in CRO-colonized HSCT patients: the historic group *vs*. the study group; **(G)** CRO-targeted therapy effective rate in CRO-colonized HSCT patients: the historic group *vs*. the study group; and **(H)** mortality rate in CRO-colonized HSCT patients: the historic group *vs*. the study group.

For all HSCT patients in the study group, the duration of fever resolution was shorter compared to the historic group as shown in **Figure 3A** (3.72 ± 2.27 *vs*. 3.14 ± 1.59 days, *p* = 0.025), and total duration of hospitalization was shorter compared to the historic group as shown in **Figure 3B** (30.61 ± 8.01 *vs*. 26.74 ± 4.73 days, *p* < 0.001). The effective rate of antibiotic therapy after the first FN episode and the mortality rate in the study group were 56.9% and 1.1%, respectively, compared to the historic group, which were 66.8% and 6.6% as shown in **Figures 3C, D** (*p* = 0.045, *p* < 0.001).

For CRO-colonized patients in the study group, the duration of fever resolution was also shorter compared to the historic group as shown in **Figure 3E** (3.24 ± 2.06 *vs*. 28.32 ± 5.83 days, *p* = 0.002), and the total duration of hospitalization was shorter compared to the historic group as shown in **Figure 3F** (34.18 ± 9.36 *vs*. 28.32 ± 5.83 days, *p* = 0.010). The effective rate of CRO preemptive therapy and the mortality rate in the study group were 96.6% and 2.4%, respectively, compared to the historic group, which were 76.9% and 19.4% as shown in **Figures 3G, H** (*p* = 0.041, *p* = 0.046).

### 3.7 Univariate and multivariate analyses of different factors between the historic group and the study group

To exclude the influence of differences in baseline discrepancies on the efficacy of anti-infective therapy between the two groups, univariate and multivariate logistic regression analyses and stepwise analysis were used to compare the independent factors and efficacy of anti-infective therapy between the two groups. The results showed that baseline and demographic characteristics, including gender, age, primary disease, type of transplant, gender of the donor and recipient, blood type of the donor and recipient, and HLA, were not statistically significant between the study and the control groups (**Table 3**). The maximum temperature, CRP, duration of G-CSF, duration of FN, and duration of recovery from neutropenia used at the time of hospitalization were not significantly different in both groups (**Table 3**). Multivariate analysis showed that the Xpert Carba-R screening group had a shorter length of hospitalization than the historic group (OR = 0.94, 95% CI 0.88–0.99, *p* = 0.038). Xpert Carba-R screening for HSCT patients in the study group reduced the risk of CRO-associated mortality (1.1% *vs*. 6.6%, OR = 0.12, 95% CI 0.01–0.75, *p* = 0.021).

**Table 3:** Univariate and multivariate analysis between historic group and study group.

Metrics		Historic group N (%)	Study group N (*%*)	Univariate OR (95% CI)	*P*	Multivariate OR (95% CI)	*P*	
Characteristics								
	Gender			1.05 (0.7-1.58)	0.739			
	Male	110 (55.8%)	105 (57.1%)					
	Female	87 (44.2%)	79 (42.9%)					
	Age			1.44 (0.94-2.2)	0.096			
	≤45y	144 (73.1%)	123 (66.8%)					
	>45y	53 (25.4%)	61 (30.4%)					
Primary disease				0.96 (0.64-1.44)	0.858			
	AL	64 (32.5%)	60 (32.6%)					
	Others	10 (5.1%)	7 (3.8%)					
Type of HSCT				0.75(0.43-1.32)	0.274			
	Autologous	32 (16.2%)	22 (12.0%)					
	Allogenic	165 (83.8%)	162 (88.0%)					
Gender of donor and recipient					0.133		
	Compatible	91 (46.2%)	71 (38.6%)					
	Incompatible	106 (53.8%)	113 (61.4%)					
Blood type of donor and recipient				0.68 (0.46-1.02)	0.102		
	Compatible	116 (58.8%)	93 (50.5%)					
	Incompatible	81 (41.2%)	91 (49.5%)					
HLA				0.68 (0.45-1.04)	0.073			
	Identical	84 (42.6%)	62 (33.7%)					
	Non-identical	113 (57.4%)	122 (66.3%)					
Parameters								
Max temperature(°C)		38.83±0.63	38.66±0.58	0.84 (0.73-0.96)	0.276		
Max CRP (mg/L)		129.35±49.73	119.44±49.90	0.99 (0.98-1)	0.374			
Duration of G-CSF used(d)		9.05±4.11	8.28±4.25	0.99 (0.89-1.09)	0.100		
Duration of FN (d)		11.06±4.14	10.28±4.28	0.84 (0.2-1.23)	0.102		
Duration of recovery from neutropenia (d)		7.05±4.13	6.27±4.27	0.46 (0.11-1.92)	0.112		
Duration of fever resolution		3.72±2.27	3.14±1.59	0.45 (0.21-0.95)	0.025	0.88 (0.13-1.04)	0.072
Duration of hospitalization		34.18±9.36	28.32±5.83	0.93 (0.91-0.96)	0.010	0.94 (0.88-0.99)	0.038
Antibiotic therapy after first FN episode				0.42 (0.18-0.98)	0.045	0.75 (0.28-1.99)	0.559
	Effective	112 (56.9%)	123 (66.8%)					
	Ineffective	85 (43.1%)	61 (33.2%)					
Survival status					<0.001	0.12 (0.01-0.75)	0.021
	Survival	184 (93.4%)	181 (98.9%)					
	Death	13 (6.6%)	2 (1.1%)					

AL, acute leukemia; HSCT, hematopoietic stem cell transplantation; CRP, C-reactive protein; G-CSF, granulocyte colony-stimulating factor. FN febrile neutropenia; HLA, human leukocyte antigen. Neutropenia, ANC <0.5×109/L.

## 4 Discussion

Highly sensitive and rapid detection of CRO colonization or infection is a critical issue for clinicians to provide guidance for infection control activities and for drugs for preemptive therapy, which is important for reducing the mortality rate in HSCT patients (Anandan et al., 2015; Iovleva and Doi, 2017). This prospective study demonstrated that HSCT patients undergoing infection control measures and CRO preemptive therapy based on the results of Xpert Carba-R screening and/or culture had a significantly shortened length of hospitalization and decreased mortality. Surveillance for CRO colonization can aid healthcare institutions in formulating timely and effective infection control measures and in preventing the spread of antimicrobial-resistant bacteria, including CRO (Sun et al., 2019).

At present, intestinal colonization with CRO has been reported in different patients, but mainly in critically ill patients, and the colonization rate can be as high as 6.8%–45% (Dautzenberg et al., 2015; Zarakolu et al., 2016). HSCT patients undergo high-dose chemotherapy and stem cell transplantation, which can reduce the richness and alter the composition of their gut microbiota. The high sensitivity of immediate RS-Carba-R means that it can promptly identify patients with CRO colonization and detect the carbapenemase genotypes they carry. In our study, a gradual decline in the monthly detection rates of CRO gut colonization by the RS-culture and/or RS-Carba-R was observed in the study group, dropping from 21.2% in the first month to 2.9% in the last month, indicating that potent active CRO colonization screening is essential for this special severe immunosuppressive population.

Schwaber et al. and Friedman et al. reported that successful interventions to decrease CP-CRO infection rates included early effective identification of CRO-colonized patients (Schwaber et al., 2011; Tacconelli et al., 2014; Friedman et al., 2017). Modifying therapeutic regimens can reduce BSI rates in neutropenic patients using rectal swab results to guide therapy (Huang et al., 2020). In our study, the prevalence of CRO-BSI in HSCT patients with CRO colonization was 25.5% in the historic group and 4.8% in the study group, which means that a PCR-based method of identifying CRO colonization is more likely to help guide therapy of CRO-related infections than traditional RS-cultures since colonization identified by the RS-culture has low sensitivity and does not provide effective information on the type of carbapenemase. The CRO infection mortality rate dropped from 19.4% to 2.4% in patients with CRO gut colonization. Forcina et al. demonstrated that through active surveillance, contact precautions, and early-targeted therapies in neutropenic carriers at the onset of fever, the cumulative incidence of CRE-BSI and septic shock at 1  year after HSCT was significantly reduced (Forcina et al., 2017). Tumbarello et al (Tumbarello et al., 2012). also demonstrated the advantage of a triple combination of colistin, tigecycline, and meropenem on day 30 mortality versus monotherapy (69.7% vs. 45.7% survival) in a retrospective analysis of 125 patients with CRKP-BSI infections. These results strongly support the key role of continuous and hypersensitive screening (e.g., PCR-based assays) for initiating an early and directed combination antimicrobial therapy in the prevention and control of CRO-BSI.

Knowledge of carbapenemase genes may be useful in making clinical decisions, including early diagnosis and treatment, which is critical for reducing morbidity and mortality due to CRO−associated infections. Xpert Carba-R has been reported to show excellent performance detecting CRO from rectal swabs in a variety of clinical specimens (Moubareck et al., 2020; Bai et al., 2021). In the meantime, more sensitive and precise surveillance and more efficient CRO preemptive therapy led to a gradual decline in detection rate and colonization rate of CRO, which suggested that the rapid and actionable screening of CP-CRO colonization, coupled with the optimization of rapid, effective CRO preemptive anti-infective strategies, has the potential to limit the spread of CP-CRO. A recent study (Zhou et al., 2024) in lung transplant recipients demonstrated that real-time CRO screening of donor organs using Xpert Carba-R prior to transplantation assisted clinicians in rapidly formulating lung transplantation strategies and reduced CRO-related donor-derived infection mortality in recipients. Multivariate analysis revealed that CRO-related mortality from donor-derived infections within 60 days was significantly lower in the Xpert Carba-R screening group compared to the control group (3.85% *vs*. 96.15%, OR = 0.05, 95% CI 0.003–0.74, *p* = 0.03).

Many of the newer β-lactam/β-lactamase inhibitor combinations are not active against organisms carrying class B metallobetalactamase enzymes, so distinguishing CRO colonization with class A or D carbapenemases from those with class B enzymes rapidly is critical (Tenover, 2021). It has been shown that using a rapid PCR test to screen for carbapenemase-producing *Enterobacterales* in rectal or stool swabs enables more efficient infection prevention and control compared to culture-based methods (Corless et al., 2020). In our study, CRO preemptive therapy—guided by the combined culture/Carba-R method—significantly shortened the duration of fever resolution and total duration of hospitalization in all HSCT patients and CRO-colonized patients. At the same time, the effective rate of CRO preemptive therapy increased, and mortality decreased.

In the study group, 83.3% (30/36) of the CRO isolate strains were carbapenemase-producing and NDM enzymes, which were the most common enzyme types, while no VIM and IMP were detected by the Carba-R assay, which is in line with the carbapenem resistance mechanism of CRO isolate strains that are prevalent in the Chinese region (Wang et al., 2018). Indeed, the rising incidence of carbapenemases other than KPC, NDM, OXA-48, VIM, and IMP has been found in certain regions of the world, such as GES, SPM, and certain IMP subtypes that were outside the scope of the commercially available Xpert Carba-R assay (Gill et al., 2020), urgently requiring the expansion of genotypic targets to improve detection scope (Garza-Ramos et al., 2015; McCracken et al., 2019; Bush and Bradford, 2020). It is recommended that the detection capabilities of the Carba-R assay be expanded, thereby increasing the global utility of the test. The research-use-only Xpert Carba-R NxG can expand the detection spectrum of the current Carba-R assay to include SPM, GES, and expanded IMP variants, which requires further in-depth investigation (Gill et al., 2020).

In conclusion, our study showed a positive effect of screening based on immediate rectal swab Xpert Carba-R for carbapenem−resistant organisms, with reductions in infection mortality in HSCT patients. It can effectively overcome drug resistance, break the chains of transmission, and aid healthcare institutions to effectively control the spread of carbapenem-resistant organisms.

## Data Availability

The raw data supporting the conclusions of this article will be made available by the authors, without undue reservation.
